# Atypical late-onset severe gastritis in immune dysregulation, polyendocrinopathy, enteropathy, and X-linked (IPEX) syndrome: 2 case reports

**DOI:** 10.1097/MD.0000000000024318

**Published:** 2021-01-22

**Authors:** Youhong Fang, Youyou Luo, Jingan Lou, Jie Chen

**Affiliations:** Department of Gastroenterology, The Children's Hospital, Zhejiang University School of Medicine, National Clinical Research Center for Child Health, 3333 Bin Sheng Road, Hangzhou 310052, Zhejiang Province, China.

**Keywords:** case report, FOXP3, gastritis, IPEX syndrome, pylorus stenosis

## Abstract

**Rationale:**

The immune dysregulation, polyendocrinopathy, enteropathy, and X-linked (IPEX) syndrome is a rare disorder that most often manifests in the early stages of life. IPEX syndrome with a late onset, presenting with severe gastritis has rarely been reported.

**Patient concerns:**

Two male adolescents presented with recurrent vomiting, severe malnutrition, and growth retardation due to severe gastritis.

**Diagnoses:**

Esophagogastroduodenoscopy of the 2 patients revealed rare presentations of severe gastritis with multiple ulcers and stenosis of the pylorus. Next-generation sequencing revealed 2 novel variants in gene *FOXP3* in the patients who were diagnosed with the IPEX syndrome.

**Interventions:**

Both patients were treated with a high calorie formular enteral nutritional therapy. In addition, the pylorus of patient 1 was enlarged by balloon dilation, while patient 2 was treated with mercaptopurine and low dose prednisone.

**Outcomes:**

Symptoms and nutritional status of the patients improved after treatment.

**Lessons:**

Chronic severe gastritis with stenosis of the pylorus could be an atypical manifestation of the IPEX syndrome. The use of next-generation sequencing is highly suitable for the diagnosis of atypical IPEX syndromes.

## 1 Introduction

The IPEX syndrome (immune dysregulation, polyendocrinopathy, enteropathy, and X-linked syndrome) is a rare immune disorder that most commonly presents in the early stages of life but can occur antenatally^[1]^ or later in life.^[2]^ The syndrome is characterized by severe enteropathy, chronic dermatitis, early-onset type I diabetes mellitus (T1DM), autoimmune thyroiditis, antibody-mediated cytopenia, and other autoimmune diseases.^[3]^ IPEX syndrome is caused by mutations in the forkhead box protein 3 (*FOXP3*) gene, which is located in the centromeric region of the X chromosome and encodes a key factor in human CD4^+^CD25^+^ regulatory T (Treg) cell development.^[4,5]^

Clinical features of the IPEX syndrome have previously been described, and most IPEX syndrome patients suffer from severe diarrhea during the first days of life. However, an increasing number of atypical cases have been diagnosed by *FOXP3* gene sequencing, indicating various manifestations of IPEX syndrome.^[6,7]^ IPEX syndrome patients who present with atypical symptoms have often been misdiagnosed before the correct diagnosis, or received the information late in life. An early diagnosis of IPEX syndrome could provide better treatment and improve patients’ quality of life. Here, we report 2 rare IPEX syndrome patients who presented with late-onset severe gastritis and pyloric stenosis, which has not been reported previously.

## 2 Ethics statement

This study was approved by the Children's Hospital of Zhejiang University, School of Medicine. Informed written consents were obtained from the patients and their parents for publication.

## 3 Case report

### 3.1 Case 1

**Figure 1:**
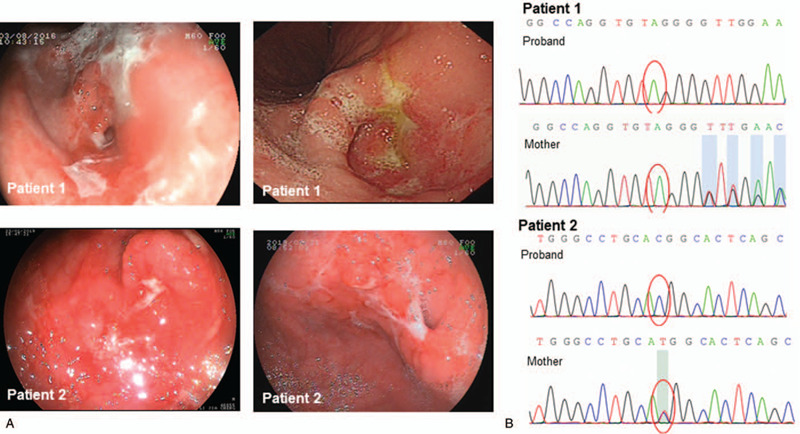
(A) EGD indicates severe pangastritis in both patients who presented with edema, erosion, and multiple ulcers of the stomach. Both the patients also showed obvious stenosis of the pylorus. (B) Mutations of the *FOXP3* gene in 2 patients. Patient 1: chrX:49107810, exon11, c.1280dupC, insertion, p.P427fs. Patient 2: chrX-49111940, exon8, c.766A>G, missense, p.M256 V (leucine zipper).

Patient 1, a 14-year 1-month-old, was the second child in his family, born at term with a birth weight of 3350 g after an uneventful pregnancy. The baseline information is shown in the Table 1. During hospitalization, the laboratory tests revealed normal level of white blood cell count and hemoglobin. His serum albumin level and his liver function were normal. The expression of antineutrophil cytoplasmic antibodies and other autoantibodies, including antinuclear antibodies, antibodies against glutamic acid decarboxylase, anti-insulin antibodies, anti-islet cells, and antibodies against tyrosine phosphatase were all negative. The lymphocyte subsets analysis was normal, while the levels of serum immunoglobulin (Ig) G, Ig A, and Ig M were significantly decreased (Table 2). The most remarkable finding of esophagogastroduodenoscopy (EGD) was a severe gastritis involving the entire stomach, especially its sinuous part (Fig. 1A). The appearance and histology findings of endoscopy are summarized in Table 2. Rapid urease testing and hematoxylin and eosin staining of the biopsy sample indicated a *Helicobacter pylori* (HP) infection.

**Table 1:** The clinical characteristics of the 2 patients with IPEX syndrome.

Patient	Sex	Onset age	Admission age	Initial symptoms	Family history	ZHFA	ZBMIA/ZWFA	Other presentations
P1	M	9Y	14Y1M	Vomiting	Unremarkable	−3.29	−3.53	Growth failure, anemia, hypo-immunoglobin
P2	M	12Y8M	13Y3M	Vomiting, diarrhea	Unremarkable	−2.69	−2.90	Autoimmune hepatitis, growth failure, hypothyroidism, anemia

**Table 2:** The clinical and laboratory characteristics of the 2 IPEX patients.

Patient	EGD	Histopathology	Colonoscopy	Serum Ig E/Ig G levels	Treg/CD3^+^CD4^+^Treg/CD3^+^
P1	Severe gastritis, multiple ulcers, stenosis of the pylorus	Infiltration of lymphocytes, plasma cells, neutrophils, and eosinophils (6–39/HP)	NA	<18.9 IU/mL1.95 g/L(5.00–10.00 g/L)	3.90% (4.52–10.98%)1.98% (2.49–6.16%)
P2	Severe gastritis, multiple ulcers and stenosis of the pylorus	Chronic inflammation, eosinophil infiltration (10–25/HPF)	Normal	<18.9 IU/mL21.8 g/L	6.87%3.66%

He received anti-HP therapy, and a repeated HP test subsequently returned negative. However, the ulcers of the stomach did not recover. Because of the stenosis of pylorus, he received balloon dilation for four times in prior years. The patient's vomiting decreased after this treatment, and he began to gain weight with enteral nutrition therapy; however, the gastritis and ulcers of the stomach did not show any significant improvement. The latest balloon dilation was performed approximately 2 years ago. Because of the persistent gastritis and low immunoglobin levels, an immunodeficiency was suspected and he had whole exon sequencing (WES) examination. WES revealed a hemizygous mutation of the *FOXP3* gene, chrX:49107810, exon11, c.1280dupC, insertion, p.P427fs (Fig. 1B). The mutation (a base shift) results in a change in the protein, which is thought to result in a loss of function. The locus of the mutation has so far not been reported. Treg/CD3^+^CD4^+^ and Treg/CD3^+^ expression was investigated and indicted low expression of Treg cells: Treg/CD3^+^CD4^+^ expression was 3.90% (reference range: 4.52–10.98%), and Treg/CD3^+^ expression was 1.98% (reference range: 2.49–6.61%). This indicated a decrease in expression of the FOXP3 protein on T cells. The patient was then diagnosed with the IPEX syndrome and prescribed mercaptopurine because of persistent gastritis. However, the patient experienced recurrent respiratory bacterial infections and therefore stopped the treatment of mercaptopurine. Due to the economic situation of the patient's family, HSCT was not an option. Despite the discontinuation of the medication, during a period of more than 3 years, the patient did not experience any symptoms. The *Z* score of height for age and body mass index (BMI) for age were both between -3 SD and -2 SD.

### 3.2 Case 2

Patient 2, a 13-year 3-month-old boy, was born as the second child of his mother. He had a healthy 17-year-old sister. Patient 2 was diagnosed with an autoimmune liver disease 3.5 years before presentation to the authors and was treated with prednisone at a dose of 1.5 mg/kg per day, which was gradually decreased to 2 mg per day. The patient had taken prednisone 2 mg per day for more than 2 years with no detectable effect on liver function. However, the patient continued to suffer from watery diarrhea and vomiting for already 7 months and was diagnosed multiple ulcers of the stomach and chronic diarrhea in another hospital. In this facility, he was treated with omeprazole and supportive treatment; however, his condition did not improve.

Upon admission, he was extremely malnourished with a *Z* score of height for age was -2.69, and the *Z* score of BMI for age was -6.20. The cervical and inguinal lymph nodes were enlarged (with the largest being 1.5 cm × 1 cm in size), and he did not have hepatosplenomegaly or edema. The laboratory investigations showed a normal white blood count and mild anemia, as well as a slight reduction in albumin, while the liver function was normal. The lymphocyte subsets and serum immunoglobulin levels of the patient were all within normal range. The expression of autoimmune diseases related antibodies, described in the first case, were all negative. His levels of serum total triiodothyronine and free triiodothyronine were decreased due to malnutrition. EGD showed severe chronic inflammation, multiple ulcers in the stomach, and pyloric stenosis, which resemble with the first case (Fig. 1A). The endoscopy and histological data of the biopsy are summarized in Table 2. The abdominal enhanced computed tomography scan indicated cirrhosis and fibrosis of the liver. Liver biopsy indicated autoimmune hepatitis. Next-generation sequencing revealed a missense mutation located at leucine zipper domain, chrX-49111940, exon8, c.766A>G, missense, p.M256V(Fig. 1B). This mutation was considered variant with an unknown effect on protein function according to American College of Medical Genetics and Genomics. The locus of the mutation had not been reported previously. His Treg/CD3^+^CD4^+^ and Treg/CD3^+^ expression was normal compared with age matched healthy children, Treg/CD3^+^CD4^+^ was 6.87%, and Treg/CD3^+^ was 3.66%. These data did not indicate a decrease in the expression level of the FOXP3 protein in the patient. Combined with the clinical presentation, he was diagnosed with IPEX syndrome.

This patient was treated with partial parenteral nutrition and oral mercaptopurine (1.5 mg/kg per day), while also continuing to take prednisone (2 mg per day). The patient's vomiting drastically improved, while the gastritis did not get much better, as indicated by the EGD repeated 1 month later. Despite this, 8 months after the beginning the above-described treatment, the patient could tolerate a normal diet, and his *Z* score of BMI for age increased from -6.20 SD to -3.97 SD. He was also advised to undergo HSCT therapy. However, he also had liver cirrhosis and his parents decided to continue the current medical therapy.

## 4 Discussion

IPEX is a primary immunodeficiency disease caused by mutations of the *FOXP3* gene, which encodes a member of the forkhead transcription factor family^[8]^ expressed primarily in CD4^+^CD25^+^ regulatory T cells, and leads to multiorgan autoimmunity in IPEX patients.^[9]^ Furthermore, other mutations in genes such as *IL2RA*, *LRBA*, *CTLA4*, *STAT1 GOF*, *STAT3 GOF*, *STAT5b*, *TTC7A*, *TTC37*, and *DOCK8* are related to IPEX-like syndrome.^[10]^ The clinical presentations of IPEX syndrome are variable and typically involve severe enteropathy, T1DM, and eczema,^[11]^ while the symptoms often overlap with other distinct clinical manifestations, including those of hematologic disease, renal disease, hepatic disease, lymphadenopathy, and severe infectious disease.^[10]^ Diarrhea usually begins within the first few months of life in the majority of IPEX patients, and some patients have histories of food allergies.^[12]^ Histologically, nearly half of the patients have villous atrophy on small bowel biopsy.^[13]^ The immunoglobulin levels are generally normal, except for elevated Ig E levels in most IPEX syndrome patients.^[10]^ CD4^+^ and CD8^+^ lymphocyte subset counts are usually normal.

More than 70 mutations have been revealed in IPEX syndrome.^[11]^ However, the severity of symptoms is unpredictable and is not related to the mutation type. In addition, patients with the same mutations could have completely different phenotypes, which could reflect the complex intracellular interactions of FOXP3.^[14]^

With awareness of IPEX syndrome and recognition of *FOXP3* gene mutations, an increasing number of atypical IPEX syndrome patients have been identified. Some patients with IPEX syndrome have mild and recurrent courses, which can delay the diagnosis.^[11]^ Notably, some patients even have gastritis along with other immune diseases.^[12,13]^ Here, we reported 2 patients with rare late-onset atypical IPEX syndrome who mainly presented similar symptoms of severe gastritis with pyloric stenosis. We previously reported an IPEX syndrome patient who had a similar presentation as these 2 patients.^[12]^ Villus atrophy was observed in the descending part of the duodenum, and pathological eosinophil infiltration was reported.^[15]^ The 2 patients had disease onset during adolescence without the typical enteropathy observed in most IPEX patients, and their EGD had similar findings with multiple ulcers throughout the stomach, especially around the sinus and the body of the stomach, along with pylorus stenosis. Patient 1 had HP infection at the first biopsy; however, after anti-HP treatment, the inflammation and ulcers did not show improvement. Moreover, the appearance of the EGD did not show a chronic infection caused by HP. Histology revealed an infiltration of multiple inflammatory cells, which did not resemble a HP infection. The serum IgG, IgA, and IgM levels in patient 1 were lower than the normal range, indicating immunodeficiency, which is not commonly observed in IPEX patients. Recently, several patients with hypogammaglobulinemia were reported; here, a functional B cell class switching defect was suggested as a possible explanation.^[16]^ Patient 2 was first diagnosed with autoimmune hepatitis in another hospital and was treated with prednisone; however, the histological investigation showed rapid deterioration, severe fibrosis, and atrophy of the liver. According to this presentation, autoimmune hepatitis could be one of the symptoms of IPEX syndrome. The mutation of patient 2 was not previously reported and was not validated by a study addressing the protein function. The level of Treg cells of this patient was normal. It has been reported that patients with missense and splicing mutations exhibit varying expression levels of the FOXP3 protein, and 2 patients with missense mutations even had markedly elevated percentages of FOXP3 expressing CD4^+^ cells. ^[10]^ According to the previously experienced symptoms and his comorbidity of other autoimmune disease, he was considered to be suffering from IPEX. To fully confirm this diagnosis, it would be good to perform a study on the FOXP3 protein function. Table 3 lists the clinical features and mutations of the *FOXP3* gene in 3 of the reported IPEX syndrome patients with gastritis. Except for 1 patient we reported previously, the other 2 patients both had early disease onset and underwent gastrectomy. The patients reported here experienced a late disease onset. Supporting our diagnosis and intervention, the patients achieved remission under the medication therapy and supportive treatment. The stenosis of pylorus may be caused by the chronic ulcers in these 2 patients.

**Table 3:** The clinical features and gene mutations of *FOXP3* of previously reported IPEX syndrome patients presenting with severe gastritis.

Disease onset	Symptoms	Mutation	Other presentations	Serum IgE levels	Treatment	Outcome	Reference
<1 Y	Recurrent ear infections	R347H (FKH domain)	T1DM, mild xerosis, pancreatic exocrine failure, growth failure	>230 IU/mL	IS/gastrectomy	Remission, followed up until 19 yrs old	Gambineri et al^[13]^
22M	Bleeding gastritis	R347H (FKH domain)	T1DM, hyperglobulinemia, respiratory infection, failure to thrive, pancreatic atrophy	Normal221 IU/mL	Omeprazole, total gastrectomy	Remission, followed up until 22 yrs old	Rodrigo et al^[17]^
3 Y	Vomiting	Lys250del (leucine zipper)	TIMD, anemia food allergy		IS, HSCT	Remission	Luo et al^[12]^

Regarding the treatment of the IPEX syndrome, there are no standard therapeutic strategies, but the main methods include immune suppression and HSCT.^[7,17]^ Normally, the effect of T cell-targeted immunosuppressant (IS) is better than that of other forms of IS. The only cure for the disease is HSCT, and it is better to perform HSCT before more organs are affected. According to the latest multicenter study, the survival rate after IS treatment was comparable to that after HSCT.^[15]^ Although most of the symptoms of the 2 patients were drastically reduced in the 2 cases described in this article, their gastritis did not recover. The previously reported patient by our team underwent HSCT, his symptoms got dramatically better, and his gastritis fully remitted. Therefore, we suggest that HSCT may be the best choice for patients with severe gastritis.

In summary, we have diagnosed 3 IPEX syndrome patients, 2 patients in the present report and 1 previously reported. The EGD of the 3 patients revealed similar extents of gastritis, which were not reported in the literature. In all 3 patients, next-generation sequencing was performed, which revealed 2 novel mutations of *POXP3*. However, the impact of these novel mutations was not confirmed by a functional study. Moreover, the mechanism that causes the gastritis in these patients remains unclear and requires further research.

Altogether, according to our experience, chronic severe gastritis with stenosis of pylorus could be an atypical presentation of the IPEX syndrome and they may be a late-onset of the disease. Next-generation sequencing could help in the diagnosis of such atypical patients.

## Author contribution

YF participated in the coordination and interpretation of data and wrote the manuscript. JC participated in the study design and helped draft the manuscript. YL and JL collected the clinical data of the patients and interpreted the results of molecular analysis. All authors read and approved the final manuscript.

**Conceptualization:** Jie Chen.

**Data curation:** Youyou Luo, Jingan Lou.

**Project administration:** Youhong Fang.

**Resources:** Youyou Luo, Jingan Lou.

**Supervision:** Jie Chen.

**Writing – original draft:** Youhong Fang.

**Writing – review & editing:** Jie Chen.

## Correction

The funding number appeared incorrectly as LQ19H030005 and has been corrected to LQ19H030010.
